# The joining of the Hsp90 and Hsp70 chaperone cycles yields transient interactions and stable intermediates: insights from mass spectrometry

**DOI:** 10.18632/oncotarget.4954

**Published:** 2015-07-22

**Authors:** Carla Schmidt, Victoria Beilsten-Edmands, Carol V. Robinson

**Affiliations:** ^1^ Department of Chemistry, University of Oxford, Oxford, UK

**Keywords:** Hsp70/90 chaperone cycle, co-chaperones, client proteins, mass spectrometry, cross-linking

## Abstract

The Hsp70/Hsp90 chaperone cycles depend on the coordinated interplay of several co-chaperones including Hsp40, Hop and peptidyl-prolyl isomerases such as FKBP52. Because of the many proteins involved in these interactions it is often difficult to delineate all possible combinations of subunits in the complexes formed. We employed mass spectrometry to monitor the assembly and to determine the favoured pathways within these chaperone cycles. Combining the subunit composition with chemical cross-linking and proteomics allowed us to define interaction interfaces, protein dynamics and new intermediates.

## INTRODUCTION

The Hsp70/Hsp90-based cellular machinery stabilises proteins for correct folding or re-folding in response to stress. It requires a cohort of co-chaperones that interact at different stages of the Hsp70/Hsp90 cycles and consequently regulate specificity for the high number of substrates. Client proteins include steroid hormone receptors, transcription factors or kinases [1-3]. A general model that has emerged over the last few decades includes binding of a client protein to the Hsp70/40 chaperones followed by transfer from Hsp70 to Hsp90 via Hop (the Hsp70-Hsp90 organising protein) [4]. Peptidyl-prolyl isomerases (e.g. FKBP52) and co-chaperones (e.g. p23) then lead to formation of the mature complexes which keep the client in an activatable state [5, 6].

Hsp40 is required to form complexes with Hsp70 and act as a catalyst to bind partially folded substrates or clients [7]. Hsp70 contains nucleotide- and substrate-binding domains which move independently prompting proposals of allosteric control mechanisms between the two domains leading to an elongated ADP conformation and a docked/compact ATP state [8, 9]. We previously characterised Hsp70 as being predominantly monomeric [10], although Hsp70 dimers have been reported in solution and crystal structures [11, 12].

Hsp90 plays a role at the later stages of the Hsp70/Hsp90 cycle. It interacts with misfolded proteins to prevent their aggregation, however, Hsp90 alone cannot refold these proteins to their native state [3]. Its main function is to stabilise client proteins and to regulate their activation with the help of numerous co-chaperones [13]. Hsp90 is almost exclusively dimeric. It contains a C-terminal dimerisation domain, a middle domain and an N-terminal nucleotide-binding domain, connected by a charged linker [14].

Recent developments have made mass spectrometry (MS) a powerful tool in structural biology. MS allows the determination of subunit stoichiometries, interaction modules and the topology of protein complexes [15]. Significantly for this research it enables the analysis of dynamic equilibria and heterogeneous protein assemblies, such as chaperone cycles [16]. We applied MS to study the dynamic complexes of the Hsp70/Hsp90-chaperone machinery. Prevalent intermediates in both cycles were identified and a final client-transfer complex containing the glucocorticoid receptor (GR) was defined. This final client-transfer complex not only contained the anticipated Hsp90 dimer but also contained an unexpected Hsp70 dimer. Addition of the immunophilin p23 to this client-transfer complex induced the transfer of the client from Hsp70 to Hsp90 preparing it for its further action and eventual transfer to the nucleus [17]. We propose that the Hsp70 dimer forms within the stable intermediate complex, as the two chaperone cycles meet to facilitate handover of client proteins from Hsp70 to Hsp90.

## THE HSP-90 CYCLE

We first explored the heterogeneity of the Hsp90 complexes formed in the presence of the co-chaperones Hop, FKBP52 and Hsp70 [10]. Hop is a crucial interaction partner of Hsp90 facilitating client transfer from Hsp70 to Hsp90 [4]. By incubating equimolar amounts of Hsp90 and Hop we found that (Hsp90)_2_(Hop)_1_ is the predominant complex although binding of a second Hop was also observed albeit at low intensities. Likewise, incubation with the immunophilin FKBP52 [18] led to the formation of (Hsp90)_2_(FKBP52)_1_ and (Hsp90)_2_(FKBP52)_2_ complexes [10].

**Figure 1:**
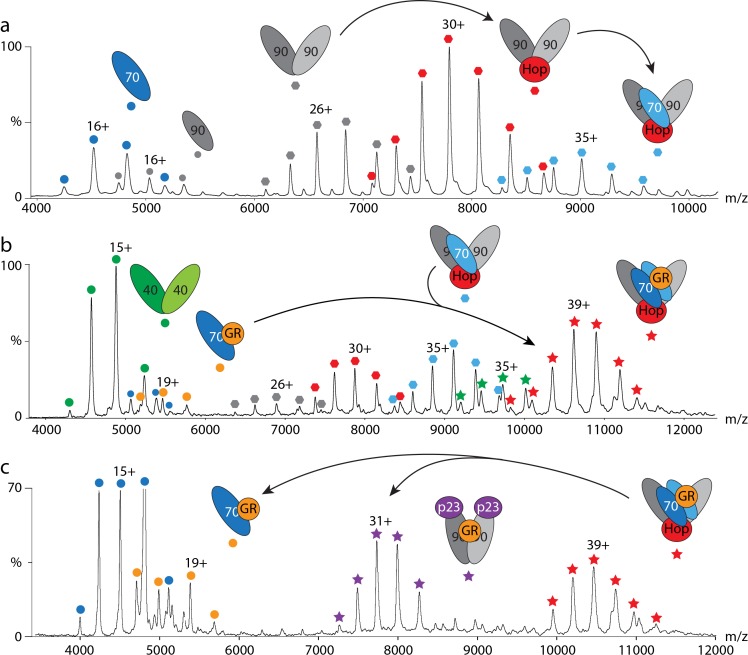
Hsp70/Hsp90 cycles followed by mass spectrometry **a.** Hsp90 requires prior binding of Hop to interact with Hsp70. **b.** The Hsp70 and Hsp90 cycles join after binding of GR to Hsp70 in the presence of Hsp40 (orange circles) and pre-assembly of the (Hsp90)_2_(Hop)_1_(Hsp70)_1_ complex (blue hexagons). A stable client-transfer complex containing an Hsp70 dimer is formed (red stars). **c.** Addition of p23 leads to disassembly of the client-transfer complex (red stars) yielding a new complex: (Hsp90)_2_(GR)_1_(p23)_2_ (purple stars).

We next challenged the Hsp90/Hop complexes with different amounts of FKBP52; one Hop could readily be exchanged by FKBP52. The (Hsp90)_2_(Hop)_1_ complex was also observed and was more prevelant than its analogue (Hsp90)_2_(FKBP52)_1_. A control experiment revealed no interactions between Hop and FKBP52 alone, confirming that Hop and FKBP52 compete for binding sites on Hsp90. Interactions between Hsp90 and Hsp70 were not observed when incubating the proteins alone. However, in the presence of equimolar amounts of Hop a (Hsp90)_2_(Hsp70)_1_(Hop)_1_ complex formed (Figure 1a) underlying the importance of Hop in facilitiating this interaction. Addition of FKBP52 to this intermediate led to a chaperone assembly of the composition: (Hsp90)_2_(Hsp70)_1_(Hop)_1_(FKBP52)_1_.

These experiments allowed the calculation of K*_D_* values for the binary interactions and the generation of a model of the Hsp90 cycle. In this model, dimeric Hsp90 can bind one copy of either Hop or FKBP52 and both intermediates can bind another copy of the same or the other co-chaperone. Furthermore, Hsp70 cannot bind Hsp90 without prior binding of Hop to Hsp90. K*_D_* values calculated from these reactions give insights into the favoured binding events at the cellular concentrations of chaperone proteins [10].

## AN HSP70 DIMER STABILISED BY POST-TRANSLATIONAL MODIFICATIONS

Client binding to Hsp70 is facilitated by the co-chaperone Hsp40 [7, 19]. We therefore first probed interactions between Hsp70 and Hsp40. After incubation in solution we did not observe Hsp70/40 complexes. However, in the presence of the client protein GR, we observed a stable (Hsp70)_1_(GR)_1_ complex indicating that interactions with Hsp40 are transient and of a catalytic nature. Indeed, incubating Hsp70 with GR alone did not result in binding of the client, but Hsp40 and GR clearly formed an (Hsp40)_2_(GR)_1_ intermediate complex [17].

We then considered the transfer of the GR client from Hsp70 to Hsp90. For this we first investigated complex formation of Hsp70, Hop and Hsp90 without client protein. We found that unmodified Hsp70 incorporated one Hsp70 molecule into the intermediate complex (Hsp90)_2_(Hop)_1_(Hsp70)_1_, while post-translationally modified Hsp70 integrated as an Hsp70-dimer [17]. Including GR with this cohort of proteins showed that prior binding to Hsp70 in the presence of Hsp40 is necessary for its insertion into this complex (Figure 1b).

## JOINING THE HSP70 AND HSP90 CYCLES

**Figure 2:**
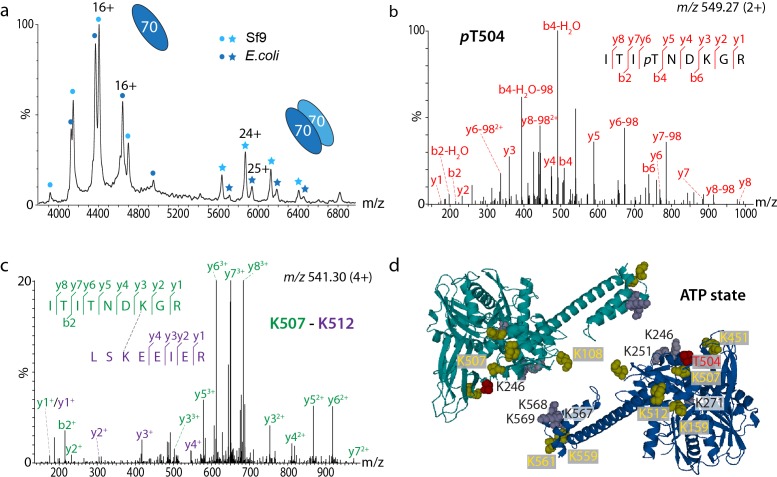
The Hsp70 dimer is stabilised by post-translational modifications **a.** Post-translationally modified Hsp70 (expressed in Sf9 insect cells) shows a higher population of dimer than unmodified Hsp70 (expressed in *E. coli*, uniformly labelled with heavy-isotopes to induce a mass shift in the mass spectrum). **b.** MS/MS spectrum of the phosphosite (pT504). **c.** Cross-linked di-peptides were identified by MS/MS. **d.** The dimer interface is stabilised by acetylation sites (yellow) and the phosphosite (red) which, together with additional lysines (grey), lead to a charge-driven association perturbed by increasing ionic strength.

The presence of an Hsp70 dimer in the client-transfer complex was surprising and contradictory to previous studies [20]. We therefore combined advanced MS with site-directed mutagenesis to probe this Hsp70 dimer interface [17]. MS experiments revealed the presence of an Hsp70 dimer that was stabilised by phosphorylation and acetylation (Figure 2a). Using proteomics we identified seven acetylation sites and one phosphosite (Figure 2b), the latter at a known hotspot for phosphorylation conserved in several eukaryotic species [21]. We probed the significance of this phosphosite by generating phosphomimic variants of Hsp70 and found that they showed increased dimerisation. We also incubated phosphorylated Hsp70, and the phosphomimic variants, with a phosphatase. We found that the intensity of the phosphorylated Hsp70 dimer was reduced, while that of the phosphomimic was stable. Together these observations allow us to conclude that phosphorylation is important for the stability of the Hsp70 dimer.

To define the dimer interface we used chemical cross-linking to probe interactions in phosphorylated Hsp70 in the presence of ATP (Figure 2c). We considered a number of high-resolution structures to model our cross-linking data and found that both the ATP and ADP states of the *E. coli* homolog DnaK (PDB IDs 2KHO and 4B9Q) best accommodate the majority of our cross-links implying that the two conformations readily interconvert in solution (Figure 2d). Two cross-links identified in the presence of nucleotides could not be assigned to intra-subunit cross-links and therefore defined the Hsp70 dimer interface in both ATP and ADP conformations. We also employed a comparative cross-linking strategy described previously [22] to compare dimerisation of phosphorylated and non-phosphoforms of Hsp70 and to compare dimer formation in the presence of ATP and ADP. Comparative cross-linking revealed that ATP and ADP states exist in a dynamic equilibrium and that dimerisation is anti-parallel in both cases.

Having established the dimer interface we projected our post-translational modifications onto high-resolution structures. Since we found that the acetylation and phosphorylation sites align at the dimer interface, this implies stabilisation by ionic interactions. An increased ionic strength in the incubation buffer was found to perturb this dimerisation interface. To rule out dimerisation in a substrate-binding manner, i.e. by recognition of the inter-domain linker of one Hsp70 molecule by the substrate binding domain of a second Hsp70 molecule, we generated a variant of Hsp70, which is not able to bind a substrate (V438F [23]). Interestingly, however, a large population of this variant was able to dimerise ruling out this mechanism of association.

The resulting client-transfer complex was defined as (Hsp90)_2_(Hsp70)_1_(Hop)_1_(GR)_1_ or (Hsp90)_2_(Hsp70)_2_(Hop)_1_(GR)_1_ depending on the status of Hsp70 post-translational modifications. We further employed chemical cross-linking to define the arrangement of the subunits within this complex. We observed a number of inter-protein cross-links verifying that two Hsp70 molecules are present in the final complex. Our cross-links also positioned the TPR-binding motifs of Hop in upward- and downward-facing arrangements as suggested previously [24] and located GR close to Hsp90. We then added the co-chaperone p23 to promote client transfer. The mass spectra revealed formation of a new complex, (Hsp90)_2_(p23)_2_(GR)_1_, without Hsp70 or Hop present (Figure 1c), confirming successful handover of the client [17].

## SUMMARY AND OUTLOOK

**Figure 3:**
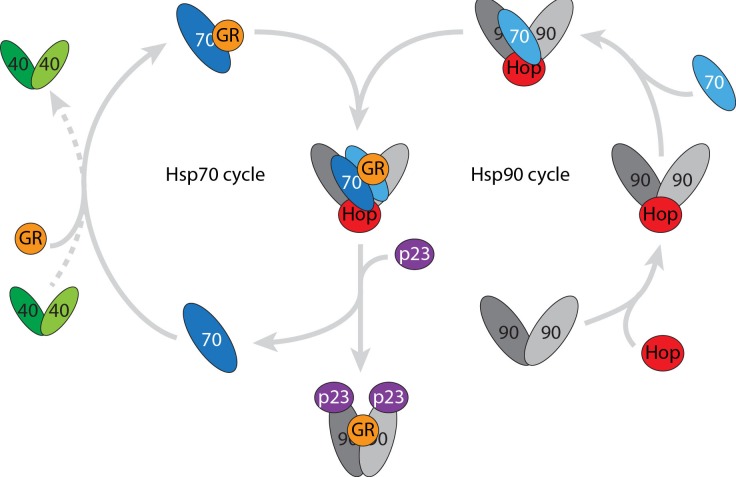
A stable intermediate is formed when the Hsp90 and Hsp70 cycles come together Catalytic quanitities of dimeric Hsp40 interact with the client (in this case the ligand binding domain of the GR) and promotes formation of the (Hsp70)_1_(GR)_1_ complex. The Hsp90 dimer interacts with Hop and monomeric Hsp70 prior to forming the stable transfer complex (Hsp90)_2_(Hsp70)_2_(Hop)_1_(GR)_1_. This transfer complex is stabilised by post-translational modifications however the client is readily transfered to Hsp90 when co-chaperone p23 is added.

In this research perspective we highlight the power of advanced mass spectrometry to investigate heterogeneous and dynamic protein assemblies. In summary we not only observed the established catalytic role of Hsp40 during substrate binding to Hsp70 but also in the dimerisation of Hsp70. Anti-parallel Hsp70 dimers have been proposed previously in yeast [11] and *E. coli* [12] but their interactions and their functional roles have not been ascribed. The client-transfer complex identified here, containing an anti-parallel Hsp70 dimer, forms when one Hsp70 monomer associates with an Hsp90 dimer, and the second Hsp70 brings the client to the transfer complex (Figure 3). The remarkable stability of this transfer complex was unexpected given that it readily proceeds to transfer the client to Hsp90 once challenged with the co-chaperone p23, suggesting its functional relevance *in vivo*. As Hsp90 also plays a role in tumour growth, by stabilising essential proteins, prevention of Hsp70 dimerisation and association with Hsp90 suggests new avenues for therapeutic interventions in cancer.
